# Properties of a New Insulation Material Glass Bubble in Geopolymer Concrete

**DOI:** 10.3390/ma14040809

**Published:** 2021-02-08

**Authors:** Noor Fifinatasha Shahedan, Mohd Mustafa Al Bakri Abdullah, Norsuria Mahmed, Andri Kusbiantoro, Sam Tammas-Williams, Long-Yuan Li, Ikmal Hakem Aziz, Petrică Vizureanu, Jerzy J. Wysłocki, Katarzyna Błoch, Marcin Nabiałek

**Affiliations:** 1 Center of Excellence Geopolymer and Green Technology (CEGeoGTech), Universiti Malaysia Perlis (UniMAP), P.O. Box 77, D/A Pejabat Pos Besar, Kangar 01000, Perlis, Malaysia; Fifinatasha@unimap.edu.my (N.F.S.); norsuria@unimap.edu.my (N.M.); ikmalhakem@gmail.com (I.H.A.); 2 Faculty of Engineering Technology, Universiti Tun Hussein Onn (UTHM), Parit Raja 86400, Batu Pahat, Johor, Malaysia; andri@uthm.edu.my; 3 Faculty of Engineering and Technology, Liverpool John Moores University, Cherie Booth Building, Byrom St., Liverpool L3 3AF, UK; S.E.TammasWilliams@ljmu.ac.uk; 4 School of Marine Science and Engineering, University of Plymouth, Plymouth PL4 8AA, UK; long-yuan.li@plymouth.ac.uk; 5 Faculty of Materials Science and Engineering, “Gheorghe Asachi” Technical University, 700050 Iasi, Romania; peviz@tuiasi.ro; 6 Department of Physics, Częstochowa University of Technology, 42-201 Częstochowa, Poland; wyslocki.jerzy@wip.pcz.pl (J.J.W.); katarzyna.bloch@wip.pcz.pl (K.B.)

**Keywords:** glass bubble, geopolymer concrete, thermal conductivity, specific heat, thermal diffusivity

## Abstract

This paper details analytical research results into a novel geopolymer concrete embedded with glass bubble as its thermal insulating material, fly ash as its precursor material, and a combination of sodium hydroxide (NaOH) and sodium silicate (Na_2_SiO_3_) as its alkaline activator to form a geopolymer system. The workability, density, compressive strength (per curing days), and water absorption of the sample loaded at 10% glass bubble (loading level determined to satisfy the minimum strength requirement of a load-bearing structure) were 70 mm, 2165 kg/m^3^, 52.58 MPa (28 days), 54.92 MPa (60 days), and 65.25 MPa (90 days), and 3.73 %, respectively. The thermal conductivity for geopolymer concrete decreased from 1.47 to 1.19 W/mK, while the thermal diffusivity decreased from 1.88 to 1.02 mm^2^/s due to increased specific heat from 0.96 to 1.73 MJ/m^3^K. The improved physicomechanical and thermal (insulating) properties resulting from embedding a glass bubble as an insulating material into geopolymer concrete resulted in a viable composite for use in the construction industry.

## 1. Introduction

Current research endeavours are heavily invested in addressing energy and environmental issues due to the global emphasis on sustainability [1,2]. Buildings are energy-intensive structures made from construction materials, the production of which is generally hazardous to the environment [3]. Buildings are regarded as shelters and designed for thermal comfort, especially in regions with harsher climates [4,5], and the thermal fluctuations that are frequent occurrences in these regions resulted in increased energy consumption per building via its respective internal thermal regulation systems (air-conditioners and heaters) [6,7]. Passive insulation systems are seen as a less energy-intensive option for buildings in harsher climates, which has the net benefit of decreasing energy costs and being eco-friendly [8,9].

The thermal (insulating) properties of concrete buildings are crucial for environmental sustainability via respective energy consumption [10,11]. The improvement of buildings’ thermal insulating properties could help regulate their internal temperatures and minimise their (energy-intensive) heating and cooling systems. However, improvements of thermal insulating properties usually come at the cost of the concretes’ physicomechanical performance, making up the buildings [12,13,14,15,16,17]. The improvement to thermally insulating properties while maintaining the concrete’s physicomechanical properties can be realised via the use of novel geopolymer concretes embedded with a glass bubble acting as its thermal insulation material. The thermal conductivity, specific heat, and thermal diffusivity of the thermal insulation materials embedded within the geopolymer concrete would enhance the residential and commercial buildings’ energy efficiency (Hu, Wang, & Ge (2012)) [10]). A material is regarded as an excellent insulating material is its thermal conductivity and diffusivity are low, and its specific heat high, properties that are especially crucial in concrete buildings situated in harsh climatic conditions.

Glass bubble has seen increasing use in structures such as polypropylene composite, epoxy-matrix composite, and cement composite in the form of fillers, additives, aggregates, and cement replacements [18,19,20,21,22,23,24]. Its spherical hollow configuration and its high crush strength, low density, ease of workability, chemical inertness, good water and oil resistance, and excellent thermal insulation properties expanded its applicability across multiple industries, one of them being the construction industry [24]. Its low density (125 kg/m^3^) and low thermal conductivity (0.044 W/mK) resulted in a net benefit to the geopolymer concrete it was embedded into to act as insulation materials. The literature on using glass bubbles as insulation materials in composites reported loadings of 0–60% [17,18,19,20,21,22,23,24,25,26]. However, these loading percentages are unguided by any known convention, and the majority of reported works appear to rely more on intuition when deciding the loading percentages of glass bubbles into the geopolymer concrete matrix.

Most of the materials used in concrete production are non-sustainable and results in the emission of CO_2_, which is a known greenhouse gas (GHG). Geopolymer concretes are recently touted as a novel environmentally-sustainable construction material with the potential to replace conventional concrete in construction [27,28,29,30,31]. The literature also reported, via works of previous researchers, that geopolymer concrete outperformed regular Portland cement concrete at an optimised mix and formulation in its mechanical stability and cost [30,31,32].

Fly-ash based geopolymer concretes have been synthesised from different power stations, resulting in substantially different particle size, morphology, and composition due to the different coal powders and combustion conditions in those plants [2,30,33,34,35,36,37,38]. Fly-ash is excellent for improving early strength and durability, rendering it suitable as the primary source materials in the synthesis of geopolymer concrete [2,33,36,37,38,39,40].

The development of geopolymer concrete using a glass bubble with low thermal conductivity at room temperature and short curing time addressed all the quality-related issues prevalent in concrete production. A composite formed from thermally insulating glass bubbles and geopolymer concrete has not been reported in the literature. Therefore, the development of the composite mentioned above is a worthwhile research endeavour in green technology, sustainability, and the development of a viable replacement to conventional concrete materials that meet current building standards.

Thermal properties such as density, thermal conductivity, specific heat, and thermal diffusivity are critical for assessing the potential energy efficiency of building materials such as concrete, which is especially relevant in the context of sustainability. Some of the preferred thermal properties include low thermal conductivity, low thermal diffusivity, and high specific heat, all of which goes to mitigate the heat loss or gained via the concrete building [5,7,8,10,41].

According to the ASTM C168-97 [42], the thermal conductivity of concrete is defined as the ratio of the heat flow rate to the temperature gradient and represents the uniform flow of heat through the concrete of unit thickness over a unit area subjected to a unit temperature difference between two opposite faces. Normally, the thermal conductivity of a normal concrete lies between 3.3–0.62 W/m/K [16,43]. Thermal conductivity is mainly related to the shape, quantity, size, and interconnection pores of the insulation material used in concrete. Yun et al. (2013) posited that concrete’s thermal conductivity is a function of the glass bubble loadings (percentages) [17]. The critical property of a thermally-insulated building is for its thermal conductivity to be as low as possible, and it is expected that the presence of thermally-insulative materials such as glass bubbles within the concrete would improve the overall thermal insulation of the building.

The specific heat is defined as the amount of heat per unit mass required to change a material’s temperature by one degree (1°). Specific heat is influenced by moisture content, temperature, concrete density, and aggregate type [9,44]. The value of the specific heat of standard concrete varies between 0.8–1.8 MJ/m^3^K [45]. Materials with high specific heat are excellent thermal storage materials [46]. Concrete with excellent specific heat capacity takes longer to absorb (for example, in harsh weather conditions) and dissipate heat [44,47,48,49].

Another important thermal property is thermal diffusivity, a measure of heat transport relative to the stored energy. Materials with high thermal diffusivities are more effective at heat transfer than heat storage. The thermal diffusivity of most concrete types is low, and due to the direct correlation between thermal diffusivity and thermal conductivity, the latter will be lower as well [50]. Low thermal conductivity divided by the high volumetric heat capacity results in a downward trend of thermal diffusivity, as described in Equation (1). Thermal diffusivity is defined as the ratio of thermal conductivity to volumetric heat capacity:

α = k/𝜌C (1)

where,

α = thermal diffusivity,
k = thermal conductivity,
*ρ* = density and
C = specific heat capacity

For a building to be energy efficient, its thermal insulation performance needs to include excellent thermal diffusivity [51]. Concrete with a low thermal diffusivity will prevent it from stabilising its temperature to match its surroundings (for example the weather), which means that a lower thermal diffusivity is preferred to buffer the temperature fluctuations during extreme or harsh conditions [52].

The previously mentioned factors and cited literature affirms the potential of the embedding glass bubble in geopolymer concrete due to its excellent thermally insulating properties and minimal effect on the resulting composite concrete’s physicomechanical properties. This study investigated the effectiveness of glass bubble as a thermally insulating material at various loading percentages in geopolymer concrete, focusing on physicomechanical characteristics such as workability, density, compressive strength, water absorption, and thermally insulating properties such as thermal conductivity, specific heat, and thermal diffusivity.

## 2. Experimental Details

### 2.1. Materials

The geopolymer concrete was prepared using fine aggregate, coarse aggregate, fly-ash, and glass bubble as thermally insulating materials. The fine aggregate used in this work was the river sand, forming 40% of the aggregates’ total mass, with sizes between 75 µm–5 mm. The coarse aggregate used in this work was gravel, forming 60% of the total mass of aggregate, with sizes between 7 mm–20 µm. The fly ash, obtained from Manjung power station, located in Lumut, Perak, Malaysia, which met the conditions set out in the ASTM C618 class F fly ash (contains a more significant combination of silica, alumina, and iron (>70%) than class C fly ash), was used as the primary material for the geopolymer’s binder [53]. The selected insulating material was the micrometre-sized glass bubble (3M. Ltd.), spherically-shaped with a light hollow sphere.

The raw material based geopolymer is the geopolymer synthesised via the activation of raw material with alkaline activator solution made from combining sodium silicate (Na_2_SiO_3_) and sodium hydroxide (NaOH) solutions. The NaOH used was in pellet form at a purity of 97%, while the Na_2_SiO_3_ consisted of 9.4% Na_2_O, 30.1% SiO_2_, and 60.5% H_2_O. Its specific gravity at 20 °C is 1.4 kg/cm^3,^ and its viscosity is 0.4 Pa s. The solid/liquid and Na_2_SiO_3_/NaOH ratios were fixed at 2.0 and 2.5, respectively, based on previous research on the optimum design of fly-ash geopolymer [54,55,56].

**Figure 1:**
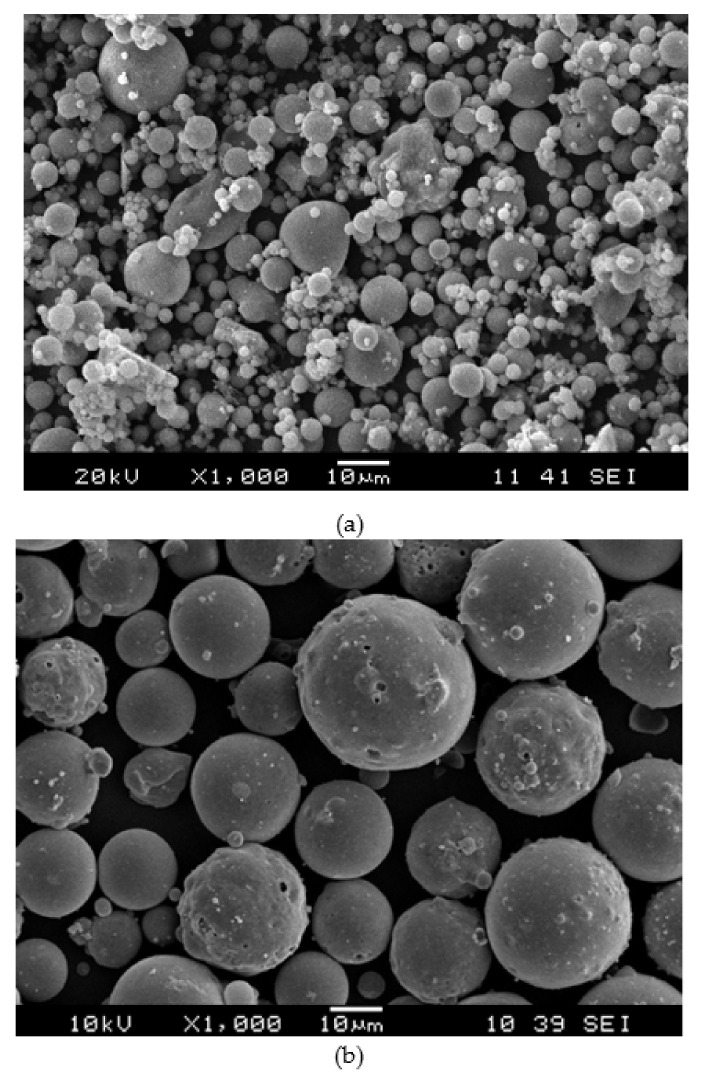
SEM micrograph analysis of raw material (**a**) fly ash and (**b**) glass bubble.

Figure 1 shows the particle morphologies observed using a JSM-6460LA model Scanning Electron Microscope (JEOL, Peabody, MA USA) for the samples coated with platinum (Pt). The majority of fly-ash particles (Figure 1a) have diameters of ~40–60 mm, while the glass bubble particle (Figure 1b) have diameters of ~60 to 80 mm. The chemical composition of fly ash and glass bubble was obtained using X-Ray Fluorescence spectroscopy (XRF) with a PANanalytical MiniPAL 4 x-ray spectrometer (Philips, Brighton, UK) and the primary composition of fly ash is SiO_2_ (55.9%), Al_2_O_3_ (28.1%), Fe_2_O_3_ (6.97%), and CaO (3.84%), while in the case of the glass bubble, its primary composition is SiO_2_ (68.5%), Na_2_O (9.90%), and ClO_2_ (15.6%).

### 2.2. Methods

The experimental works involved determining the samples’ physicomechanical properties such as its workability, density, compressive strength, water absorption, and the thermally insulating properties of the samples such as the thermal conductivity, specific heat and thermal diffusivity. The glass bubble was loaded into the geopolymer concrete as per these percentages (2.5%, 5.0%, 7.5%, 10.0%, 20.0% and 30.0%). The sodium silicate (Na_2_SiO_3_) and sodium hydroxide (NaOH) were mixed before being added to the fly ash, aggregates, and glass bubble for another 10 min. After the geopolymer concrete sample mixture was homogeneous, the fresh concrete was placed in the 100 mm mould to produce samples that would be used for density and compressive strength tests, while another batch of samples was placed in a cylinder mould (diameter = 8 cm, height = 2.5 cm) for thermal insulation tests. The samples were cured at room temperature up to the point of testing (total of 28 days). Other details about the study are tabulated in Table 1 and Table 2. 

#### 2.2.1. Workability Test

The workability tests were conducted right after mixing. The slump of the fresh geopolymer concrete was placed in a mould, and the specimens were compacted with three layers of equal volume placing and tamping using a rod, as outlined in ASTM C143 [57]. Each layer was rodded 25 times. The apparatus has a base diameter of 200 mm, a diameter of 100 mm, and a 300 mm truncated metal cone with a top. The cone was lifted upward vertically to allow the concrete sample to slump downward under the influence of gravity. The decrease in the height of the centre of the slumped concrete is called the slump. The slump was measured by placing the cone just next to the slump concrete, and the temping rod was placed over the cone so that it also covered the area of the slumped concrete. The decrease(s) in the height of concrete relative to that of the mould were recorded using a scale.

#### 2.2.2. Density Test

The density of the geopolymer concrete was measured on day 28 of curing. A cube geopolymer concrete sample measuring 100 mm on each side was first immersed in water at room temperature for 24 h, and its weight recorded as immersed weight (Wi). The sample was removed from the water and allowed to drain for a minute, then weighed, and this value was recorded as the saturated weight (Ws). After that, the sample was dried in an oven at 110 °C for 24 h, then weighed, with this weight recorded as the dried weight (Wd). Equation (2) was used to calculate the density of the sample:

(2) $$\mathrm{Density},\;D\;=\;\left(\frac{\mathrm{Wd}}{\mathrm{Ws}-\mathrm{Wi}}\right)\;\text{×}\;1000$$

The measured density and thermal conductivity can be explained using the general rule of mixtures from Voigt and Reuss model, which can predict various properties of concrete or composite materials. Certain properties of a particular composite depend only on its constituents’ relative concentrations and properties, and the mixture rule can accurately predict properties such as density and thermal conductivity using information from the glass bubble itself and the geopolymer concrete without glass bubbles. The results of these predictions are the upper-and lower-bound values of these properties, which can be calculated using Equations (3) and (4):

(3) $${\left(\frac{f}{\rho_{f}}\;+\;\frac{1-f}{\rho_{m}}\right)}^{-1}\leq \rho_{c}\;\leq \;f\rho_{f}+\;\left(1\;-f\right)\;\rho_{m}$$

and

(4) $$f\;=\;\left(\frac{V_{f}}{V_{f}\;+\;V_{m}}\right)$$

where *ρ*_*f*_ is the glass bubble density, *ρ*_*m*_ is the geopolymer concrete’s density, *f* is the volume of friction of the concrete, *V_f_* is the volume friction of the glass bubble, and *V_m_* is the volume friction of the concrete. The experimental value of the density, *ρ*_*c*_, is expected to lie between the theoretical upper and lower bounds calculated using the rule of mixture.

#### 2.2.3. Compressive Strength Test

The compressive test was carried out as per the ASTM C 39 using the previously mentioned 100 mm cube samples [58]. The samples’ straight-edged surface were cleaned thoroughly before the experiment to remove any loose sand grains and ensure that the surface is free from any other contaminants. The test began with the application of the loads onto the samples. After that, the samples were carefully placed below the centre of the compressive machine’s upper bearing block. Finally, before recording the values, the data required by the compressive machine, which are size, weight, and a specified rate of loading of the samples were adjusted. The total maximum recorded values indicated by the testing machine can be used to calculate the compressive strength of the samples using Equation (5):

(5) $$F_{m}\;=\;\frac{P}{A}$$

where

Fm = Compressive strength (MPa)
*P* = Total load (N)
*A* = Area of loaded surface (mm^2^)

#### 2.2.4. Water Absorption

The water absorption test was conducted based on the ASTM C1585-04 to obtain the volume of absorbed water by the sample upon immersion in water [59]. The weights of the samples were recorded before and after being immersed in water. The water absorption value was calculated using Equation (6).

(6) $$Water\;absorption=\;\frac{Ws-Wd\;}{Wd}\;\times \;10$$

where

*Ws* = saturated weight (g)
*Wd* = dry weight (g)

#### 2.2.5. Thermal Insulation

Thermal insulation measurement technology has seen immense improvements and advances over the years, making it useful and even crucial for understanding the principles of heat flow via insulating materials in various applications. Thermal insulation is commonly quantified using steady-state and transient methods, the former is primarily suitable for analysing materials with low or average thermal conductivities at moderate temperatures, while the latter measures the temperature-time response of the sample when a signal is sent out to create heat in a body [46,54,55,56,57,58,59,60,61]. Previous research used transient methods, or more specifically, the Transient Plane Source (TPS) technique to determine thermal properties such as the thermal conductivity, diffusivity and heat capacity of building materials at room temperature [41,47,56].

A recently developed thermal measurement technique utilising the hot strip method is called the transient plane source (TPS) technique, or the hot disk (HD) or Gustafsson probe, proposed by Gustafsson [46]. The transient measurement consists of the hot wire technique, which is mostly used for quantifying the thermal conductivity of solid non-electrically conducting materials, including cementitious materials. Relative to the steady-state methods, this technique keeps the temperature constant, which guarantees accurate measurements [41,47,56,62]. The testing is performed by recording the voltage/resistance variations when the plane sensor element is electrically heated (electrical current pulse) [63]. The value of its electric resistance *R*(*t*) in the sensor can be represented as a function in terms of the average temperature increase ∆*T*(*t*) of the sensor element in the first approximation in Equations (7) and (8), as:

(7) $$R\left(t\right)=R_{O}\left[1\;+\;\alpha \Delta \left(t\right)\right]$$

*R*_O_ is the resistance of the TPS element at time zero, *α* is the temperature coefficient of resistance (TCR) for the TPS element (at room temperature), and ∆*T*(*t*) is the mean value of temperature rise in the TPS-element due to a constant current pulse given by:

(8) ΔTt=POπ32 αkfτ

where

$$\theta =\;\frac{\alpha^{2}}{K}$$

*P*_O_ is the total output of the sensor, α is the radius of the sensor, *k* is the thermal conductivity of the sample, *τ* is the dimensionless time, *θ* is the characteristic time, and κ is the thermal diffusivity of the sample material, (*τ*) is dimensionless time function representing the energy collected effect during the time [0, *τ*], it is related to the number of sources in the sensor. The transient (dynamic) methods can be used for measuring thermal diffusivity of good-conducting solid materials, thermal conductivity, or both, for a broader range of temperatures and thermal properties [62]. 

**Figure 2:**
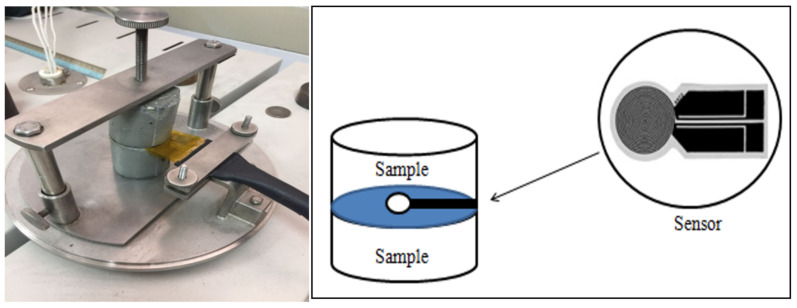
Schematic diagram of the Transient Plane Source (TPS) methods.

The TPS method or thermal constant analyser consists of various transient plane source probes connected to a computerised control unit measure, which respond as a signal and is sent out to create heat in the sample. This method adheres to the ISO standard (ISO22007-2). These methods are distinguished mainly by quick measurements (100–120 s) within −100 to 1000 °C and can discern thermal conductivities between (0.016–6 W/mK), thus making it suitable for various materials [46,56]. The TPS sensor was placed between two halves of the concrete cube sample in the configuration shown in Figure 2. After an equilibration time of ~45 min in a laboratory nominally maintained at 23 °C, measurements were obtained at a power of 0.3 W for 10 s.

This method is generally quicker when it comes to reaching the required equilibrium for obtaining the results relative to other methods, especially in determining the thermally insulating properties for concrete [61].

## 3. Results and Discussions

The samples’ physicomechanical properties such as its workability, density, compressive strength, and water absorption will be discussed based on the optimal loadings of the glass bubble in the geopolymer concrete that satisfies the minimum strength requirement of the load-bearing structure. The thermal (insulating) properties determined in this study were the thermal conductivity, specific heat, and thermal diffusivity, which will also be discussed within the abovementioned context.

### 3.1. Investigation of the Novel Geopolymer Concrete Performance of the Effects of Replacement of Glass Bubble

#### 3.1.1. Workability

**Figure 3:**
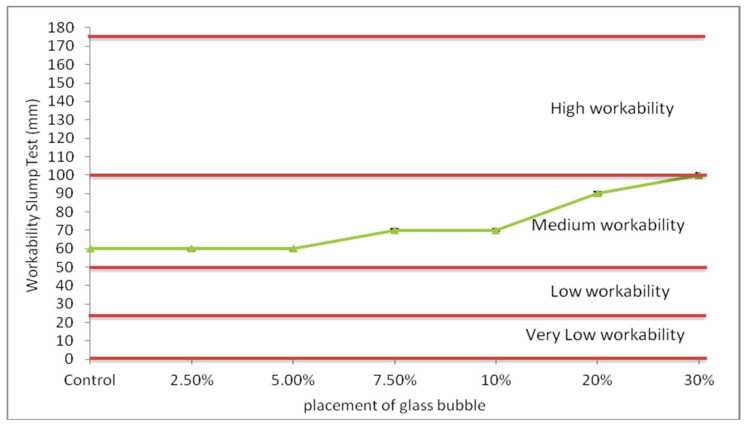
Measured slumps of geopolymer concrete with percentage placement of glass bubble (0, 2.5, 5.0, 7.5, 10, 20, and 30%).

The workability of the glass bubble loaded geopolymer concrete is shown in Figure 3. The slump values of the geopolymer concrete glass bubble loadings (0, 2.5, 5.0, 7.5, 10, 20, 30%) were found to be 60–100 mm, which translates to a value increment of ~16% relative to its unloaded (with glass bubble) counterpart.

It is evident in Figure 3 that the presence of the glass bubble within the geopolymer concrete significantly affected its workability. The change can be attributed to the increasing solid portion of the glass bubble within the geopolymer concrete, which led to increased viscosity and decreased flow of the fresh mixture via increased particulate friction, causing medium slump (suitable for a common purpose such as reinforced concrete compacted by vibrating poker, or manually). However, it should also be pointed out that the spherical configuration of the fly-ash and glass bubble particles and its fineness also influenced the concrete’s workability [35]. The shape minimises the friction of the particles with the binder, which resulted in a ball-bearing effect at the point of contact and allowing the concrete to move freely, slightly increasing the workability of the geopolymer concrete via increased loading of the glass bubble, as posited in Ranjbar, Behnia, Alsubari, Moradi Birgani, & Jumaat, (2016), and Bogas et al., (2013) [64,65].

#### 3.1.2. Density

**Figure 4:**
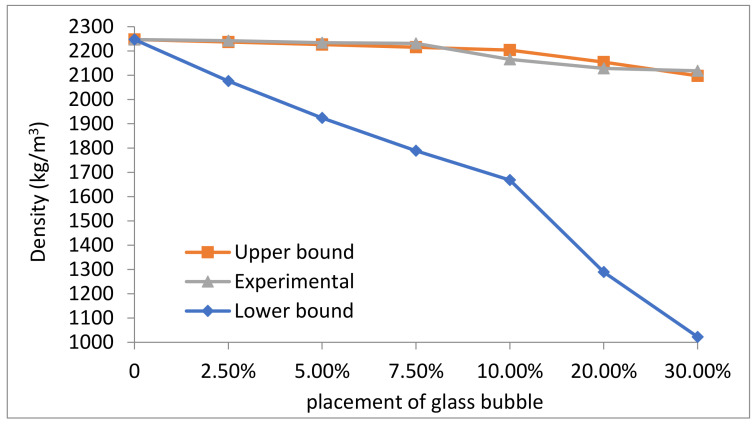
Measured density (experimental) with the lower and upper bound assumption for geopolymer concretes with different percentage placement of glass bubble.

The presence of the glass bubble in the geopolymer concrete decreased its overall density (Figure 4). The upper and lower bounds calculated (theoretical) using the rule of mixture were compared with the experimentally determined densities.

The decrease in the overall density of the geopolymer concrete due to the presence of glass bubble was also accompanied with decreases in air void or bubbles generated by air entrainment of the closed-shell structure, as concrete density is dictated by the volume fractions of its constituent materials and the respective volume of voids present within the concrete. These are some of the factors that make the geopolymer concrete’s density vital for its thermal properties, as a density of 100 kg/m^3^ could theoretically result in a thermal conductivity decrease of 0.04 W/mK [66].

**Figure 5:**
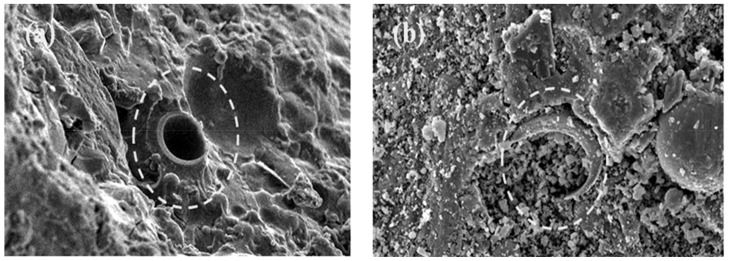
SEM image of cross-section geopolymer concrete (**a**) 2.5% placement of glass bubble, (**b**) 30% placement of glass bubble.

The experimental values were not in agreement with the lower bounds or minimum values of the calculated (theoretical) density, where the former resulted in the categorisation of the geopolymer concrete as a lightweight (lightweight concrete < 2000 kg/m^3^). The lightweight nature of the geopolymer concrete could be indicative of the fact that in the case of some percentages (the gap between lower bound to the experimental line are 7, 13, 19, 22, 39 and 50% for the geopolymer concrete with glass bubble loadings of 2.5, 5.0, 7.5, 10.0, 20.0 and 30.0% respectively), the glass bubble reacted with the geopolymer binder as the main composition of the glass bubble is silica (Si), which is also the main components involved in the geopolymerisation process. This assumption is supported by the images shown in Figure 5.

#### 3.1.3. Compressive Strength

**Figure 6:**
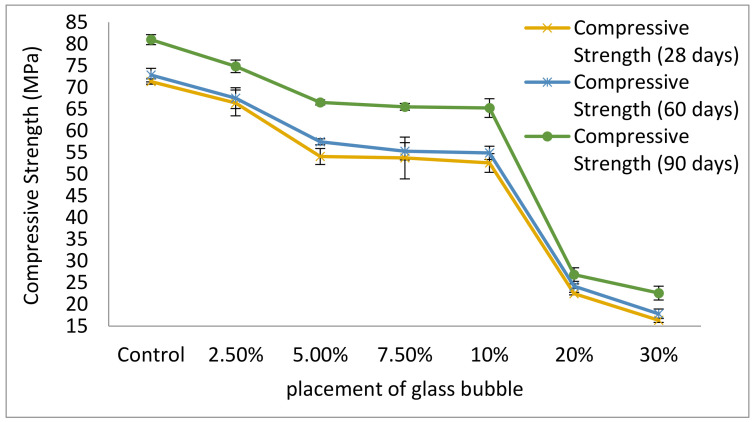
Compressive strength of geopolymer concrete with percentage placement glass bubble (0, 2.5, 5.0, 7.5, 10, 20, and 30%) with an increment of ageing days.

The effect of glass bubble loadings (0, 2.5, 5.0, 7.5, 10, 20, and 30%) on the compressive strength based on effect of aging days (28, 60, and 90 days) has shown in Figure 6. A general decrease of the geopolymer concrete’s compressive strength is evident, from its highest at 71.4 MPa (zero loadings of glass bubble) to its lowest at 16.4 MPa (at a 30% loading of glass bubble).

As per the results shown in Figure 6, it is evident that the glass bubble loading is inversely related to the compressive strength of the geopolymer concrete. This observation can be attributed to the fact that the geopolymer concrete matrix holding the glass bubble decreased, which results in the matrix hold upon the glass bubble weakening without deformation caused by the compressive load. This internal occurrence translated into decreased compressive strength on the part of the geopolymer concrete. This is also in accordance to a reported work in the literature involving the loading of glass bubble into normal concrete, where the former, at 10%, 15% and 30%, resulted in corresponding strengths of 36.2 MPa, 25.9 MPa, and 13.9 MPa, respectively [17,19]. It can therefore be surmised that the geopolymer concrete matrix also helps maintain the strength of the concrete.

Most studies involving insulation posited that the density, compressive strength, and thermal conductivity are main factors dictating the viability of the geopolymer concrete of insulation applications [14,67,68,69], therefore, determining the compressive strength of the geopolymer concrete serves as an essential checkpoint for gauging the viability of the geopolymer concrete for insulation application and makes it essential to determine the compressive strength of the composite in insulation applications.

#### 3.1.4. Water Absorption

**Figure 7:**
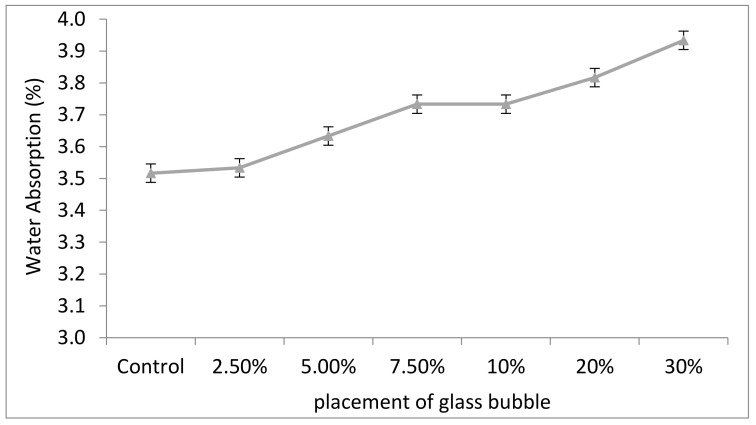
Comparison of water absorption between geopolymer concrete with percentage placement of glass bubble (0, 2.5, 5.0, 7.5, 10, 20, and 30%).

Figure 7 shows the effect of loading of glass bubble (0, 2.5, 5.0, 7.5, 10, 20, and 30%) with respect to the percentage of water absorption for 28 days. Water absorption represents the primary mechanism for water and water vapour transport in concrete where commonly lower water absorption characteristics of concrete will increase the structure’s lifespan.

The water absorption percentage slightly increased but remained within a comparable percentage of 3–6.5% of standard concrete. A slight increase of water absorption can be attributed to the increase in the glass bubble content in the geopolymer concrete, leading to decreased aggregates in the system. It also contributed to the spherical configuration that developed into capillary voids, making transportation in the geopolymer concrete possible.

### 3.2. Thermal Insulation Performance of the Novel Geopolymer Concrete with Glass Bubble

#### 3.2.1. Thermal Conductivity

**Figure 8:**
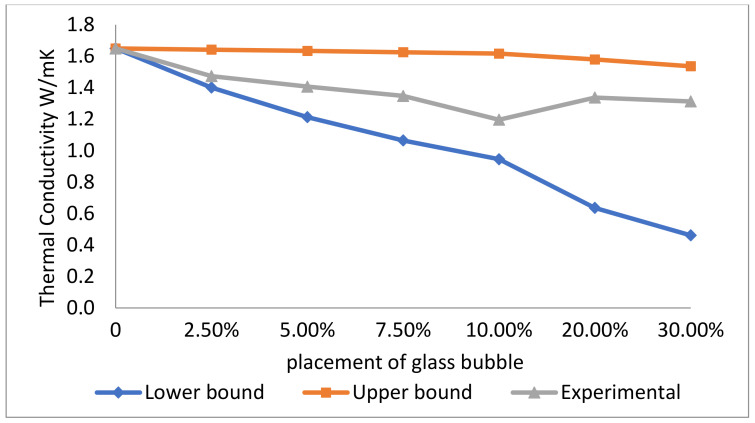
Measured thermal conductivities (with standard deviations indicated by error bars) for geopolymer concretes with different percentage placement of glass bubble with the lower and upper bound assumptions.

Figure 8 shows the thermal conductivity of geopolymer concrete for different loadings of the glass bubble. It can be seen that the control geopolymer concrete sample has a thermal conductivity of 1.65 W/mK and a density of 2247 kg/m^3^. The general trend in the plot seems to be an inverse relationship between glass bubble loading and thermal conductivity, where the thermal conductivities of 1.47, 1.41, 1.35, 1.19, 1.34, and 1.31 W/mK corresponded to 2.5%, 5.0%, 7.5%, 10.0%, 20.0%, and 30.0% glass bubble loading, respectively, resulting in an overall decrement of 20.44% in thermal conductivity. It is also evident in Figure 8 that the measured values of the thermal conductivities lie between the upper and lower bounds of the (calculated) theoretical values, which was expected.

The results confirmed that the geopolymer concrete’s effective thermal conductivity decreases with increasing loading of the glass bubble (inverse relationship). The thermal conductivity of the glass bubble itself helps decrease the overall thermal conductivity of the geopolymer concrete. The thermal conductivity of the gas within the glass bubble is 0.04 W/(mK) [26] and its solid wall has a thermal conductivity of 1.03 W/(mK) [70]. The presence of an interior gas core in the glass bubble and its increasing concentration within the geopolymer concrete will nominally decrease the thermal conductivity of the latter due to the presence of small-closed pores in the former mitigating air convection [63,64,71,72], and this effect of decreasing thermal conductivity is amplified with increasing concentrations (loadings) of glass bubble within the geopolymer concrete [73].

#### 3.2.2. Specific Heat

**Figure 9:**
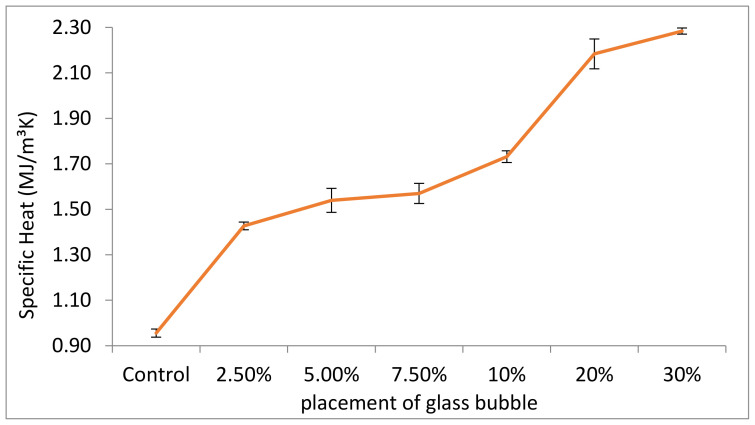
Measured specific heat (with standard deviations indicated by error bars) for geopolymer concretes with different percentage placement of glass bubble.

The measured specific heat of control (zero loadings of glass bubble) was 0.95 MJ/m^3^K for 2247 kg/m^3^, and 2.28 MJ/m^3^K for 2118 kg/m^3^ relative to its 30% glass bubble loading counterpart, which translated into a 58% decrease in specific heat at a 5.7% decrease in density (Figure 9).

**Figure 10:**
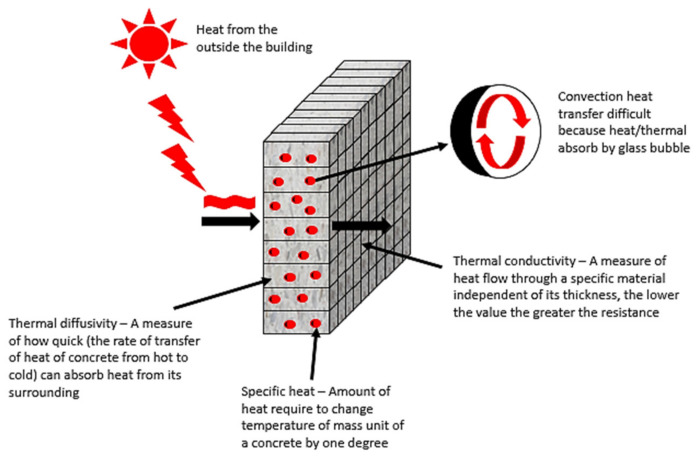
The illustration of convection heat and the function of thermal insulation properties.

The specific heat of the geopolymer concrete increased with increasing loading of glass bubble due to the presence of an interior gas core in the glass bubble, which increases the thermal storage capacity that delays the heat transfer or convection in the thermal insulation system (illustrated in Figure 10), where there is no convection if the particle size is less than 4 mm. The mean particle size (X50) of the glass bubble used in this study is 66.68 µm, and glass bubble loading leads to high specific heat, which improves the passive energy storage system, benefitting the building owner and energy supplier. Building materials with high specific heat do not need constant heat supply, due to the high specific heat materials taking longer to store and dissipate heat to its surroundings, which keeps the buildings warmer longer [74]. Also, as a raw material, fly-ash has little influence on the specific measured heat [41] than standard concrete, which only achieved specific heat at 1.0 (MJ/m^3^K) at the same density.

#### 3.2.3. Thermal Diffusivity

**Figure 11:**
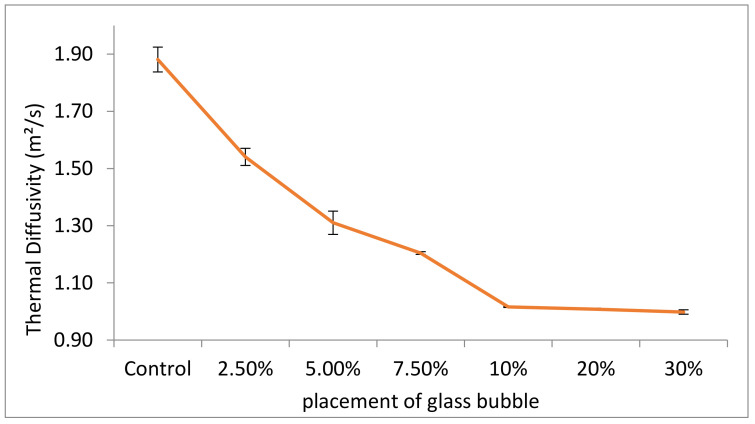
Measured thermal diffusivity (with standard deviations indicated by error bars) for geopolymer concretes with different percentage placement of glass bubble.

The measured thermal diffusivity at 30% loading of glass bubble (thermal diffusivity = 0.99 m^2^/s) decreased by 6% relative to the control sample (thermal diffusivity = 1.88 m^2^/s). Both the thermal diffusivity and density are inversely related (Figure 11).

The results indicated that diffusivity is inversely related to glass bubble loading, which could be due to the closed pore in the glass bubble delaying the temperature increase to stabilise itself vis-à-vis its surrounding temperature [75,76]. Density also influenced the thermal diffusivity of the geopolymer concrete, and consequently, the insulation behaviour [77], which can be attributed to the decreased thermal conductivity, decreasing the thermal diffusivity. This could be an offset by the decrease in density, which is necessary to decrease the thermal conductivity [78]. The measured thermal diffusivity is influenced by the thermal properties of the constituents of the concrete and is an essential factor for the control of the temperature of the building in concrete elements. As the thermal conductivity relies on the concrete’s pores, it can also be surmised that thermal diffusivity is correlated with the ratio between thermal conductivity and volumetric heat capacity.

Bouguerra et al. (2001) confirmed that the thermal conductivity and diffusivity decreases while the heat capacity increases with an increasing volume fraction of thermal insulation materials in building materials [60].

## 4. Conclusions

This paper reported the physicomechanical and thermal insulation properties of a new insulation material, the glass bubble, embedded in geopolymer concrete to enhance the latter’s thermal insulation while also preserving its functional physicomechanical properties. The physicomechanical properties such as the workability, density, compressive strength, and water absorption, and thermal insulation properties such as the thermal conductivity, specific heat, and thermal diffusivity of the geopolymer concrete embedded with glass bubble were determined and analysed.

The workability of the samples were determined to be 70 mm, its density 2165 kg/m^3^, its compressive strengths (based on curing days) 52.58 MPa (28 days), 54.92 MPa (60 days), and 65.25 MPa (90 days), and its water absorption at 3.73% at a 10% glass bubble loading, which was determined to be the most suitable configuration that satisfies the minimum strength requirement adequate for load-bearing structures. The decreases in the thermal conductivity (1.47 to 1.19 W/mK) and thermal diffusivity (1.88 to 1.02 mm^2^/s) due to the increase of void ratio from the glass bubble and the thermal conductivity of the glass bubble itself significantly improved the thermal properties of the concrete. The presence of an interior gas core of glass bubble resulted in high specific heat (0.95 up to 1.73 MJ/m^3^K) of the geopolymer concrete, leading to increased heat absorption and longer heat storage times before heat dissipation to its surroundings.

Therefore, the potential of using glass bubble as a thermal insulation material was affirmed due to its low thermal conductivity, low thermal diffusivity, high specific heat, and comparable concrete performance vis-à-vis the standard recommended strength for concrete applications.

## Figures and Tables

**Table 1:** The details parameter of the geopolymer concrete.

Materials		Parameter	
Aggregate	Coarse 60%	Aggregate	60%
	Fine 40%		
Fly Ash		Binder	40%
Sodium Silicate			
Sodium Hydroxide			
Solid/Liquid		2.0	
Na_2_SiO_3_/NaOH		2.5	
Glass Bubble (%)		0, 2.5, 5.0, 7.5, 10, 20, 30	

**Table 2:** The details mix proportion of the geopolymer concrete.

Glass Bubble (%)	Coarse Aggregate (kg/m^3^)	Fine Aggregate (kg/m^3^)	Fly Ash (kg/m^3^)	Sodium Hydroxide (kg/m^3^)	Sodium Silicate (kg/m^3^)
0	864	576	640	91.40	228.60
2.5	842.4	561	624	89.12	222.89
5.0	820.8	547.2	608	86.83	217.17
7.5	799.2	532.8	592	84.55	211.46
10.0 20.0 30.0	777.6 691.2 604.8	518.4 460.8 403.2	576 512 448	82.26 73.12 63.98	205.74 182.88 160.02

## Data Availability

The data presented in this study are available on request from the corresponding author.
